# Impact of Vaccination Time on Anti-SARS-CoV-2 Antibody Levels in Adults: A Quasi-experimental Study

**DOI:** 10.7759/cureus.71505

**Published:** 2024-10-15

**Authors:** Vibha Gangwar, Manish Verma, Arvind K Singh, Jyotsana Agarwal, Rashmi Kumari, Jaya Garg, Vinita Shukla, Anumesh K Pathak, Rajani Bala Jasrotia, Sarita Kumari

**Affiliations:** 1 Department of Physiology, Dr. Ram Manohar Lohia Institute of Medical Sciences, Lucknow, IND; 2 Department of Community Medicine, Dr. Ram Manohar Lohia Institute of Medical Sciences, Lucknow, IND; 3 Department of Microbiology, Dr. Ram Manohar Lohia Institute of Medical Sciences, Lucknow, IND; 4 Department of Biochemistry, Dr. Ram Manohar Lohia Institute of Medical Sciences, Lucknow, IND

**Keywords:** antibody titer, circadian rhythm, india, sars-cov-2, vaccination time

## Abstract

Background and aim: Vaccination time may provide an opportunity to advance immunogenicity in terms of the immune system's circadian nature. This analytical study was planned to determine the impact of forenoon and afternoon administration of the COVID-19 vaccine to adults on the magnitude of antibody response.

Method: A total of 33 healthy adults with no history of COVID-19 infection or any other disease participated in the study. They were allotted a forenoon (900-1200 hours) or afternoon (1200-500 hours) slot for vaccination. They were categorized as a forenoon or afternoon group, with 16 subjects in the forenoon and 17 in the afternoon group. With the consent of the participants, a blood sample was collected before vaccination, 30 days after the first and 30 days after the second dose of vaccination from all the subjects. The antibody titer response was measured using a commercial semi-quantitative assay, SARS-CoV-2 IgG II.

Results: The baseline antibody titer against COVID-19 was 51.41 ± 22.22 AU/mL and 53.21 ± 15.67 AU/mL in the forenoon and afternoon groups, respectively, which increased to 15773.00 ± 3231.41 AU/mL and 12970.82 ± 7608.00 AU/mL after 30 days of the first dose of the COVID-19 vaccine in the forenoon and afternoon groups, respectively. This further increased to 37007.00 ± 1697.75 AU/mL and 38012.00 ± 14001.16 AU/mL after 30 days of the second dose of the COVID-19 vaccine in the forenoon and afternoon groups, respectively. There was no difference in antibody response in subjects with forenoon and afternoon vaccinations. There was no significant difference in antibody titer in males vs. females. The study reported that antibody titers decreased with increasing age and BMI of participants.

Conclusion: The time of the day of vaccination does not impact the immune response to COVID-19, but age and BMI are important factors to consider during vaccination against SARS-CoV-2 in the adult population.

## Introduction

Millions of people are globally affected by the coronavirus disease 2019 (COVID-19) epidemic. It is a highly infectious disease caused by the severe acute respiratory syndrome coronavirus 2 (SARS-CoV-2), which has prompted a scramble for safe and effective treatments [1]. Implementation of vaccines is a valuable strategy to reduce morbidity and mortality from a variety of diseases and to prevent further outbreaks, including COVID-19 [2]. We are still focused on researching vaccines and finding new uses for licensed medicines. Reviewing the current knowledge of chronobiology as applicable to other viral pathogens may help us realize opportunities to harness the power of the circadian rhythm for SARS-CoV2.

Physiological functions of the human body exhibit a 24-hour pattern in their variations, known as the circadian rhythm. The circadian rhythm is an organism's internal clock that responds to environmental changes. This concept was first introduced by Halberg et al. in 1959 to describe this intrinsic physiological regularity [3,4]. The internal master clock is located in the suprachiasmatic nucleus of the hypothalamus, which is connected to a network of peripheral molecular clocks present in cells of virtually every tissue and organ [5]. The works of molecular clock are highly conserved, tightly regulated, and exhibit a high degree of redundancy. Multiple studies suggest that the circadian rhythm can control an organism's susceptibility to viral infection and the potency of its immunological response to pathogenic assaults [6-10]. Additionally, the severity of viral infection is dependent on the time of day at which the pathogen is encountered [7]. Therefore, the circadian clock system and circadian deviation of several immune activities may facilitate the regulation of vaccine efficacy based on the time of day. A study conducted on the hepatitis vaccine from Birmingham suggests that morning vaccination is associated with an enhanced antibody response in men [11]. One cluster randomized study observed that morning vaccination with the inactivated influenza vaccine resulted in higher antibody responses than afternoon vaccination in adults older than 65 years [12].

Previous research has shown that the key protein that is accountable for SARS-CoV-2 and host interaction is associated with the circadian rhythm (approximately 30%) [13]. Therefore, time-stamped vaccination throughout the day would enable us to quickly scale up to define the impact of time in observational studies, even though the strategy for utilizing chronobiology principles to improve the efficacy of the vaccine response against SARS-CoV-2 is multi-layered. A few studies have been conducted to demonstrate the effect of vaccination timing against COVID-19 on antibody response, which were designed as cross-sectional studies and revealed mixed results [9,14,15]. Therefore, this study was designed with a cohort design to evaluate the antibody response to COVID-19 vaccination after the first and second doses of the vaccine.

## Materials and methods

Ethical statement

The study was approved by the Ethics Committee of Dr. Ram Manohar Lohia Institute of Medical Sciences, Lucknow (ICE no. 29/22 dated 23/04/2022). The procedure was conducted in compliance with the Declaration of Helsinki. All participants were briefed about the study, and informed consent was obtained.

Study design and recruitment

It was an analytical study performed over a period of one year. Healthy subjects who came for COVID-19 vaccination at the vaccination center of Dr. Ram Manohar Lohia Institute of Medical Sciences, Lucknow, India, were recruited for the study.

The sample size was calculated assuming a mean clinically significant difference of a minimum of 1000 in geometric mean titer (GMT) (approximately 3%) of IgG after 20-30 days of the second dose with an SD of approximately 1000. We needed to enroll a minimum of 16 participants in the forenoon and afternoon groups each to be able to reject the null hypothesis that the mean GMT of IgG is equally distributed in both groups with a confidence interval of 95% and a power of 80%. Since the study involved follow-up of participants and we expected high dropouts, we doubled the sample size and enrolled 32 participants in each group.

Healthy subjects aged between 18 and 29 years were included in the study. Subjects suffering from acute infection, a history of chronic medical illness, or any immunological disorder were excluded from the study. Adult subjects were randomly chosen and contacted a day earlier from the list of the subjects who booked the slots for vaccination on the CoWIN app for COVID-19 vaccination at the vaccination center of the institute each day. Those who fulfilled the inclusion and exclusion criteria determined on the phone and consented to the study were included. They were allocated a forenoon or afternoon slot for vaccination. Following all these criteria, a total of 64 people were enrolled in this study, with 32 subjects in the forenoon and afternoon groups each.

Study protocol

The vaccine used at the vaccination center of Dr. Ram Manohar Lohia Institute of Medical Sciences, Lucknow, at the time of the study was COVISHIELD/Oxford/AZ-ChAdOx1, which is a recombinant, replication-deficient chimpanzee adenovirus vector that encodes SARS-CoV-2 spike glycoprotein. All the subjects were given a questionnaire to fill out to record their demographic variables. The subjects of the morning group were instructed to take both doses of the vaccine before 12 noon, and the subjects of the evening group were instructed to take the same after 12 noon. Their blood sample was collected before administering the first dose of the vaccine, 30 days after the first dose, and 30 days after the second dose of vaccine administration. Their IgG antibody titer response to a specific antigen of SARS-CoV-2 was tested using a commercial semi-quantitative assay for SARS-CoV-2 IgG II (Abbott Healthcare Pvt. Ltd., India) by chemiluminescent microparticle immunoassay (CMIA) for the semi-quantitative detection of IgG antibodies against the spike protein receptor-binding domain (RBD).

Statistical analysis

Mean, standard deviation, and p-value were analyzed for all the recorded variables. Baseline titers were entered as covariates. The time of the day of vaccination was entered as a fixed factor. The chi-square test and Student's t-test were used to compare the groups based on the time of vaccination: before noon (17 participants) and afternoon (16 participants). A p-value <0.05 is considered statistically significant. The Pearson correlation was used to analyze the correlation of antibody titer with demographic factors.

## Results

A total of 64 participants were enrolled for the study over a period of one year at Dr. Ram Manohar Lohia Institute of Medical Sciences, Lucknow, India. The participants were divided into two groups based on the time of vaccination. Group I consisted of 32 participants who were enrolled and followed up for COVID-19 vaccination in the forenoon (before 12 noon). Group II consisted of 32 participants who were enrolled and followed up for COVID-19 vaccination in the afternoon (after 12 noon). Out of all the subjects enrolled, 17 participants from Group I (forenoon) and 16 participants from Group II (afternoon) completed the study.

Table 1 summarizes the general characteristics of the participants. The male-to-female ratio was 14:19. There was no significant difference in gender distribution between the two groups (p = 0.88).

**Table 1:** General characteristics of the study population The chi-square test was used to compare the groups. A p-value of <0.05 is considered statistically significant. SD: standard deviation; BMI: body mass index.

Variables	Total (n=33)	Forenoon (n=17)	Afternoon (n=16)	p-value
Gender				
Male	14 (42.42%)	7 (41.17%)	7 (43.7%)	0.88
Female	19 (57.58%)	10 (58.83%)	9 (56.3%)	-
Age (years)				
Mean ± SD	27.94 ± 12.56	23.41 ± 6.61	32.75 ± 15.55	0.030
Systolic pressure (mmHg)				
Mean ± SD	125.03 ± 13.15	122.71 ± 13.41	127.86 ± 17.78	0.24
Diastolic pressure (mmHg)				
Mean ± SD	81.06 ± 9.16	79.47 ± 8.59	83.00 ± 9.79	0.27
BMI (kg/m^2^)				
Mean ± SD	34.88 ± 7.08	32.18 ± 5.74	38.17 ± 7.33	0.013

**Figure 1:**
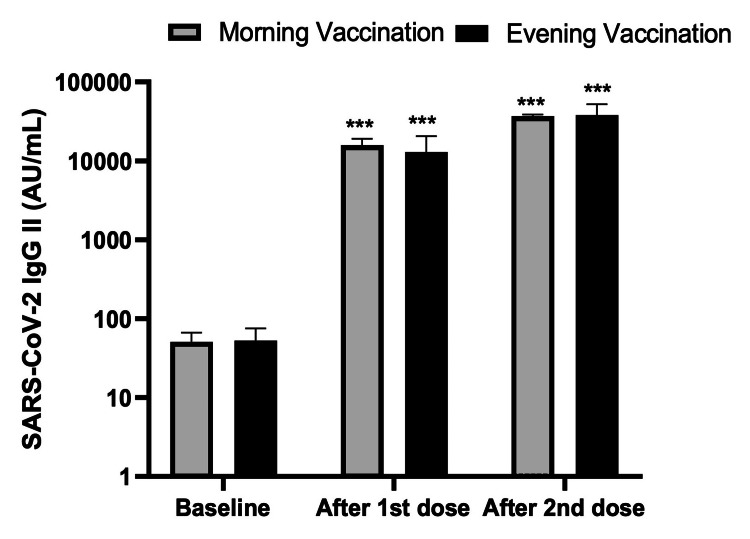
Antibody response after the first and second doses of the COVID-19 vaccine in the forenoon and afternoon groups ***p<0.0001 after comparing the first and second doses of the SARS-CoV-2 IgG II titer with the baseline titer. ANOVA test was applied for the p-value.

Figure 1 shows the antibody response after the first and second doses of the COVID-19 vaccine in the forenoon and afternoon groups. The baseline antibody titer in the forenoon and afternoon groups were 51.41 ± 22.22 AU/mL and 53.21 ± 15.67 AU/mL, respectively. The antibody titers 30 days after the first dose of the COVID-19 vaccine were 15773.00 ± 3231.41 AU/mL and 12970.82 ± 7608.00 AU/mL in the forenoon and afternoon groups, respectively. Additionally, the antibody titers 30 days after the second dose were 37007.00 ± 1697.75 AU/mL and 38012.00 ± 14001.16 AU/mL in the forenoon and afternoon groups, respectively.

Table 2 shows that the difference in the mean antibody titer before vaccination, 30 days after the first dose, and 30 days after the second dose in the forenoon and afternoon groups were not statistically significant.

**Table 2:** Association of the time of vaccination and the dose of anti-SARS-CoV-2 antibody levels Tukey's multiple comparisons test was applied. A p-value of <0.05 was considered statistically significant. ns: nonsignificant; AM: forenoon; PM: afternoon.

Combination	Δ Mean (AU/mL)	p-value
Baseline: AM Vaccination vs. Baseline: PM Vaccination	-2.000	ns
Baseline: AM Vaccination vs. After 1st dose: AM Vaccination	-15722	<0.0001
Baseline: AM Vaccination vs. After 1st dose: PM Vaccination	-12919	<0.0001
Baseline: AM Vaccination vs. After 2nd dose: AM Vaccination	-36956	<0.0001
Baseline: AM Vaccination vs. After 2nd dose: PM Vaccination	-37961	<0.0001
Baseline: PM Vaccination vs. After 1st dose: AM Vaccination	-15720	<0.0001
Baseline: PM Vaccination vs. After 1st dose: PM Vaccination	-12917	<0.0001
Baseline: PM Vaccination vs. After 2nd dose: AM Vaccination	-36954	<0.0001
Baseline: PM Vaccination vs. After 2nd dose: PM Vaccination	-37959	<0.0001
After 1st dose: AM Vaccination vs. After 1st dose: PM Vaccination	2803	ns
After 1st dose: AM Vaccination vs. After 2nd dose: AM Vaccination	-21234	<0.0001
After 1st dose: AM Vaccination vs. After 2nd dose: PM Vaccination	-22239	<0.0001
After 1st dose: PM Vaccination vs. After 2nd dose: AM Vaccination	-24037	<0.0001
After 1st dose: PM Vaccination vs. After 2nd dose: PM Vaccination	-25042	<0.0001
After 2nd dose: AM Vaccination vs. After 2nd dose: PM Vaccination	-1005	ns

**Figure 2:**
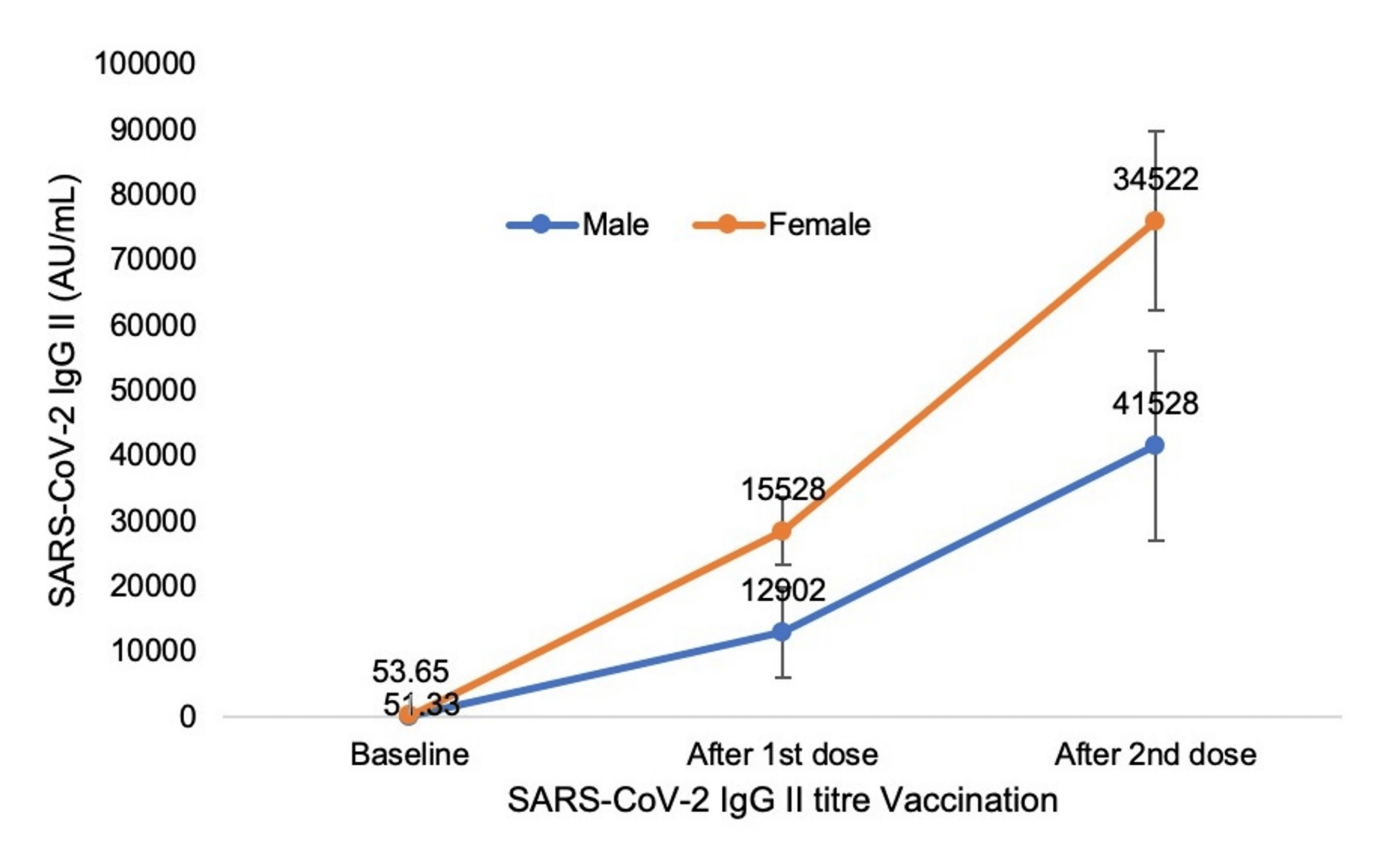
Antibody response after the first and second doses of the COVID-19 vaccine in males and females

Figure 2 shows the gender difference in the antibody response 30 days after the first dose and 30 days after the second dose of the COVID-19 vaccine. The antibody titer after the first dose of COVID-19 vaccination was 12901.75 ± 6894.53 AU/mL and 41527.93 ± 14573.42 AU/mL, respectively, in males, while 15528 ± 5108.83 AU/mL and 34522 ± 16143.37 AU/mL, respectively, in females. The difference in antibody titer in males and females after the first and second doses was not statistically significant (p = 0.57 and 0.19, respectively).

**Figure 3:**
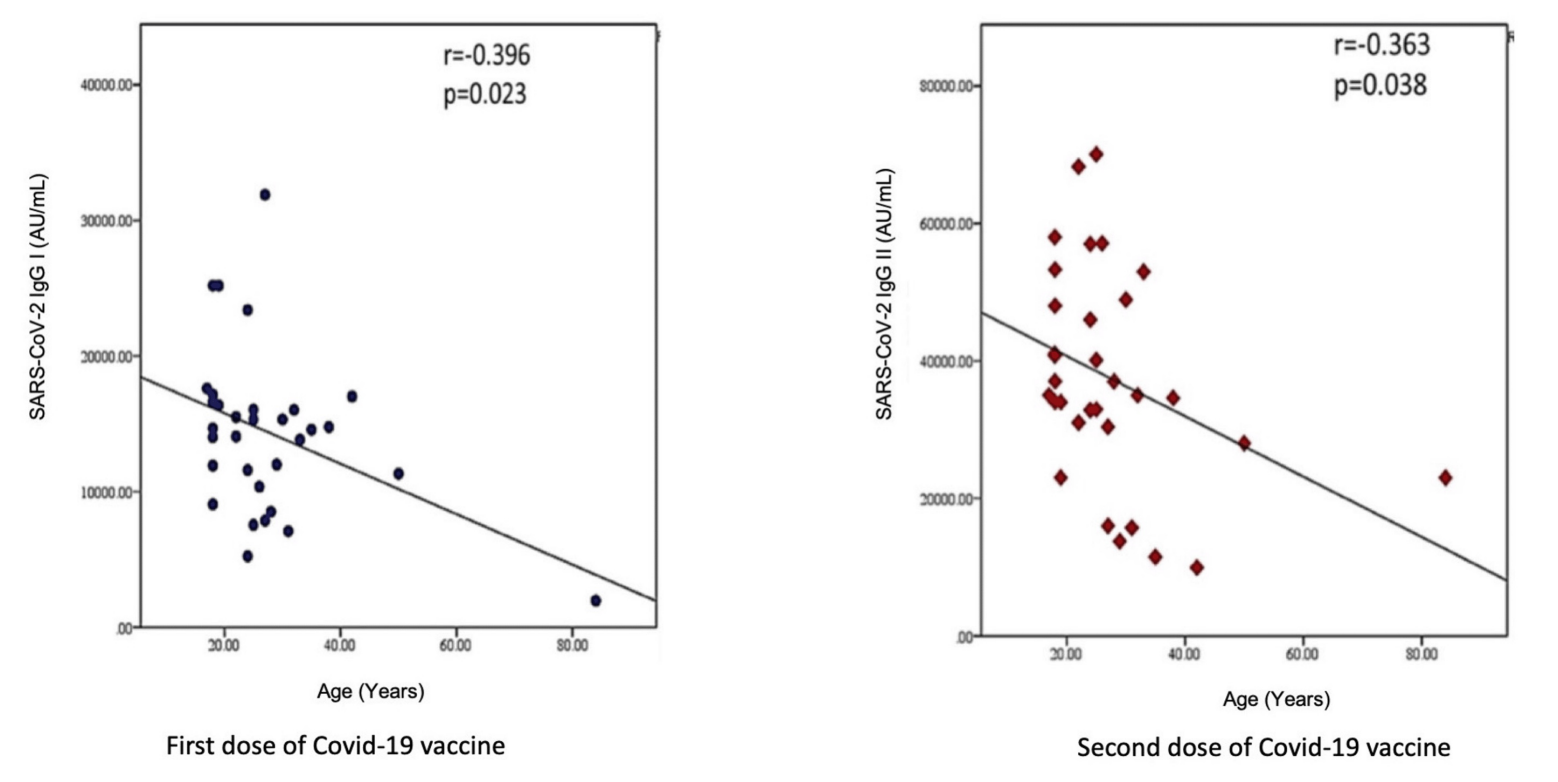
Correlation of antibody titers with age Pearson correlation coefficient (r > 0.2 considered as correlation). A p-value of < 0.05 was considered statistically significant.

Figure 3 shows the correlation of antibody titer with the participants' age. The antibody titer decreased significantly with increasing age, both after the first and second doses of the COVID-19 vaccine.

**Figure 4:**
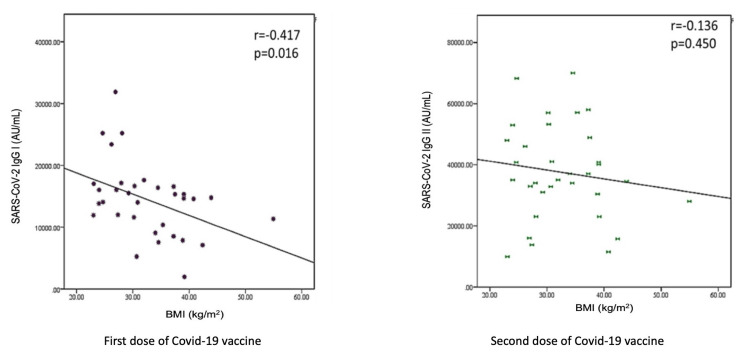
Correlation of antibody titers with BMI Pearson correlation coefficient (r > 0.2 considered as correlation). A p-value of <0.05 was considered statistically significant. BMI: body mass index.

Figure 4 shows the correlation of antibody response with the body mass index of the participants. The antibody titer significantly decreased after the first dose of the vaccine, but the correlation was not statistically significant after the second dose.

BMI was identified as a significant factor associated with the antibody titer over time (afternoon), as shown in Table 3. The univariate analysis indicated a significant association of BMI with time (β = 0.352, p = 0.052), which was also confirmed by the multivariate analysis (β = 0.443, p = 0.035). These findings highlight the influence of BMI on immune response.

**Table 3:** Regression analysis of dosing time (forenoon and afternoon) as an independent factor BMI: body mass index.

Variables	Univariate		Multivariate	
	β-coefficient	p-value	β-coefficient	p-value
Age	-0.058	0.756	-	-
BMI	0.352	0.052	0.443	0.035
SARS-CoV-2 IgG II at Baseline	-0.151	0.401	-	-
SARS-CoV-2 IgG II at Dose-2	-0.225	0.152	-	-

Table 4 shows the participants' forenoon and afternoon antibody response after the first dose of COVID-19 vaccination in the younger vs. older groups. The antibody titer after the first dose of COVID-19 vaccination was higher in the forenoon group than that in the afternoon group in the age group between 18 and 25 years. However, the difference was not statistically significant (p = 0.33). Also, there was no statistically significant difference in the antibody titer between the younger vs. older groups in the forenoon and afternoon groups (p=0.30 and 0.91, respectively).

**Table 4:** Forenoon and afternoon antibody response in the participants after the first dose of COVID-19 vaccination in the younger vs. older groups The Student's t-test was applied. A p-value of <0.05 is considered significant.

Age Group of Participants	Antibody Response 30 Days After the First Dose of Vaccine (AU/mL)		p-value
	Forenoon vaccination (9 am to 12 noon)	Afternoon vaccination (12 noon to 5 pm)	
18-25 years	17053.77 ± 8008.99	12970 ± 4063.52	0.33
26-50 years	12699.5 ± 6197.87	13024.32 ± 3266.82	0.89
p-value (t-test)	0.30	0.91	-

Table 5 shows the participants' forenoon and afternoon antibody response after the second dose of COVID-19 vaccination in the younger vs. older groups. The antibody titer was higher in the group between 18 and 25 years than in the group between 26 and 50 years in the forenoon. However, the difference was not statistically significant (p = 0.38). Additionally, there was no statistically significant difference in the antibody titer between the younger vs. older groups in the forenoon and afternoon groups (p = 0.40 and 0.45, respectively).

**Table 5:** Forenoon and afternoon antibody response in the participants after the second dose of COVID-19 vaccination in the younger vs. older groups The Student's t-test was applied. A p-value of <0.05 is considered significant.

Age Group of Participants	Antibody Response 30 days After the Second Dose of Vaccine (AU/mL)		p-value
	Forenoon vaccination (9 am to 12 noon)	Afternoon vaccination (12 noon to 5 pm)	
18-25 years	92547.42 ± 147027.03	26566.25 ± 10967.28	0.40
26-50 years	32257.6 ± 11118.04	27821.58 ± 10530.974	0.45
p-value (t-test)	0.38	0.84	-

## Discussion

The present study examined whether the time of day of vaccination alters the antibody response against SARS-CoV-2 after the first and second doses of the mRNA-1273 vaccine in a sample from the Indian population. A few previous studies have been done in this regard, which were observational studies [9,14-16]. The current study measured the anti-spike antibodies both after the first and second doses of the mRNA vaccine. A study by Yamanaka et al. in 2022 measured antibody response only after the first dose of the vaccine [15].

Our analysis revealed no significant effect of the forenoon and afternoon vaccination times on antibody response after the first and second doses of the vaccine. This finding was in accordance with previous research, which also did not find an association of SARS-CoV-2 antibody titer with vaccination time [15].

Many researchers have found an association between vaccination time and antibody response for the COVID-19 vaccine. Recent research studying 2784 healthcare workers immunized with the vaccine-type mRNA bnt162b2 or adenoviral AZD1222 revealed that anti-spike antibodies were higher in those who were vaccinated later in the day (p = 0.013) [14]. They studied only healthcare workers, and the dominant strain at the time of the study was alpha B.1.1.7 in December 2020 in the UK. The current study was done in 2022 in India, where the dominating strains were B.1.617.1 and B.1.617.2. This reflects the variation in the immunogenicity of various strains [17].

Another report studying a small cohort of healthcare workers immunized with an inactivated SARS-CoV-2 vaccine showed increased B-cell responses and anti-spike in the participants vaccinated in the morning (0900-1100 hours, n = 33) compared to those vaccinated in the afternoon (1500-1700 hours, n = 30) [18]. The sample size of this study was similar to our study, but it was a cross-sectional study with a very small sample size. Also, the duration of their morning and evening slots was very narrow. The current study considered the forenoon slot from 0900-1200 hours and the afternoon slot from 1200-1700 hours. The COVISHIELD vaccine used at our center is identical to the Oxford-AstraZeneca (ChAdOx1 nCoV-19) vaccine in composition and immunogenicity [19]. Inactivated whole-virus immunogen likely induced a polytypic response to a range of SARS-CoV-2-encoded proteins.

Studies on vaccines for some other diseases have been done to analyze the effect of the time of the day of vaccination on the immune response. Phillips et al. (2008) conducted a study on the hepatitis A vaccine and reported higher antibody titers in the morning than in the afternoon [11]. Long et al. (2016) conducted a randomized controlled trial on the influenza vaccine and reported a higher anti-influenza titer in the morning than in the afternoon for the H1N1A strain but not for the H3N2A strain [12]. The effect of time of day on the immune response after COVID-19 vaccination may be explained by the role of circadian component BMAL1 in regulating SARS-CoV-2 replication, as demonstrated by Zhuang et al. [9]. They revealed that Bmal1 silencing induced interferon-stimulated gene transcripts in Calu-3 lung epithelial cells. It provides a mechanism for the circadian pathway to limit SARS-CoV-2 infection.

Wang et al. did not find a significant association of anti-spike antibodies with sample collection time [14], while some other studies showed that in clinical influenza vaccine trials, the timing of collection of samples can have a major and potentially misleading influence on the study outcome [16,20]. We did not consider the time of sample collection in our study.

Our study found no significant gender difference in the antibody titer after the first and second doses of the COVID-19 vaccine. This is in contrast to the study done by Wang et al., who found a higher anti-spike response in women (p = 0.013) [14]. However, our study was in accordance with a recent observational study done on 727 patients in Iraq, which also showed no significant difference in both IgM and IgG production in male participants compared to women [21]. This discrepancy could be affected by genetic and also nongenetic local factors.

As shown in Tables 4, 5, our study did not find the anti-spike antibody response in the younger (18-25 years) and older (26-50 years) age group subjects both after the first and second doses of the vaccine. Bayram et al. (2021) reported seropositivity of 85.4%, 68.2%, and 37.5% among healthcare workers aged 18-34 years, 35-59 years, and >60 years following the first dose of the vaccine [22]. However, the linear regression analysis in our study showed a decreased antibody response with increasing age. According to Li et al. (2021), no apparent correlations between age and the concentrations of antibodies were observed after the second dose [23].

This study also analyzed the correlation of BMI with anti-spike antibodies after the first and second doses of the vaccine and found that the antibody titer decreased with increasing BMI. It was in accordance with a previous study, which also showed that a higher BMI was associated with lower titers of SARS‐CoV‐2 spike antibodies, suggesting the need for careful monitoring for vaccine efficacy in people with obesity [24]. It is worth noting that vaccination showed high efficacy, highlighting the robust nature of the host antibody response. Taken together, the findings of the present study suggested that the effect of time of the day of vaccination on immune response may depend on various factors, e.g., type of vaccine, age, gender, and BMI.

Limitations of the study

The study's limitations include a relatively small sample size and residual confounding by either unmeasured factors or variables measured with error, which may be a possibility. Furthermore, the appointment time will not be optional but will be allocated by the investigator so that it may be a little inconvenient for the participants. The effect of psychosocial factors on vaccination may produce some errors. Our study did not include the high-risk subjects and those with a history of diseases. We recommend future studies addressing these limitations. The possible cellular and molecular processes that could account for the antibody result are still unclear and require more research.

## Conclusions

The present study provides evidence that the vaccination time with SARS-CoV-2 is not associated with the SARS-CoV-2 antibody titer in the adult population. The results underscore the importance of recording the time of vaccination in clinical studies and future research. However, it is important to note that this study was conducted with a limited number of participants. For a more comprehensive understanding, it would be beneficial to replicate this study on a larger scale. Furthermore, our findings pave the way for future research to explore the association of other factors, such as age and gender, with the immune response and circadian rhythms. Such studies could provide valuable insights into personalized vaccination strategies and the role of biological rhythms in immune response.
